# Quantum supremacy: some fundamental concepts

**DOI:** 10.1093/nsr/nwy072

**Published:** 2018-07-06

**Authors:** Man-Hong Yung

**Affiliations:** 1 Shenzhen Institute for Quantum Science and Engineering and Department of Physics, Southern University of Science and Technology, China; 2 Shenzhen Key Laboratory of Quantum Science and Engineering, Southern University of Science and Technology, China

Quantum computation was envisioned by Feynman as a valuable means of solving quantum problems [1]. The question is, how do we prove the superiority of quantum computing over classical devices? A common misconception in describing the power of a quantum computer over a classical computer is that quantum bits can be prepared in a superposition of an exponential number of states, which cannot be achieved with classical bits. This statement is certainly true, but it does not tell us, specifically, what problems quantum computers can efficiently solve while classical computers fail to do so. In fact, based exactly on the technique of Feynman's path integral, the output (i.e. transition amplitude) of any quantum circuit can be calculated classically with a polynomial amount of memory (although the temporal cost would be exponential). Therefore any quantum computation can be simulated classically; the key question is about the efficiency.

Much effort has been made trying to pin down the power of quantum computers based on the machinery of the computational complexity theory. In particular, the class of computational (decision) problems that can be solved efficiently by a quantum computer is called BQP (bounded-error quantum polynomial time); the counterpart for classical computers is called P (polynomial time). Of course, BQP cannot be less than P; in principle quantum computers can simulate classical computers efficiently. The point is that the foundation of quantum computation cannot be established without a proof showing that BQP is strictly larger than P (i.e. BQP ≠ P). In this sense, this question is as interesting as the famous challenge of proving P ≠ NP (non-deterministic polynomial time).

A well known instance of an NP problem is the factoring problem; Shor's quantum algorithm is capable of solving the factoring problem in polynomial time, which is not achievable with the best classical algorithm discovered so far. However, we still cannot rigorously exclude the possibility of the existence of an efficient classical algorithm for factoring. Although again without a proof, it is commonly believed that quantum computers are unable to solve some of the NP problems. In fact, the proof of this statement (if true) provides a possible path to prove P ≠ NP.

A potentially less demanding question would be, when do we expect a quantum computer to be able to perform some well defined tasks (not necessarily related to any practical problem) that cannot be simulated with any currently available classical device, within a reasonable time? Once achieved, the status is called ‘quantum supremacy' [2]. One may ask, what about Grover's search algorithm, which provides a quadratic speedup over the classical search? Can we say that we can already achieve quantum supremacy with Grover's algorithm? The problem is that, instead of taking the large-*N* limit, quantum supremacy requires us to determine the actual number of qubits and gates that can no longer be simulated by classical computers within a reasonable time.

To elaborate further, we remark that there can be two notions of classical simulation, namely ‘strong simulation' versus ‘weak simulation'. Strong simulation refers to the cases of calculating the transition probabilities, or expectation values of observables, to a high accuracy in polynomial time. Weak simulation requires the probability distributions to be accurately reproduced, which involves ‘sampling' the different outcomes of the quantum devices. Some quantum circuits that cannot be strongly simulated with an efficient classical means may have efficient classical methods for weak simulation [3].

However, we should be careful about the accuracy requirement in the simulation tasks. For example, for many decision problems, it may be sufficient to determine the transition amplitudes to within an additive error, instead of multiplicative error. In other words, the classical simulability of quantum computational problems depends on the accuracy requirement.

**Figure 1.**
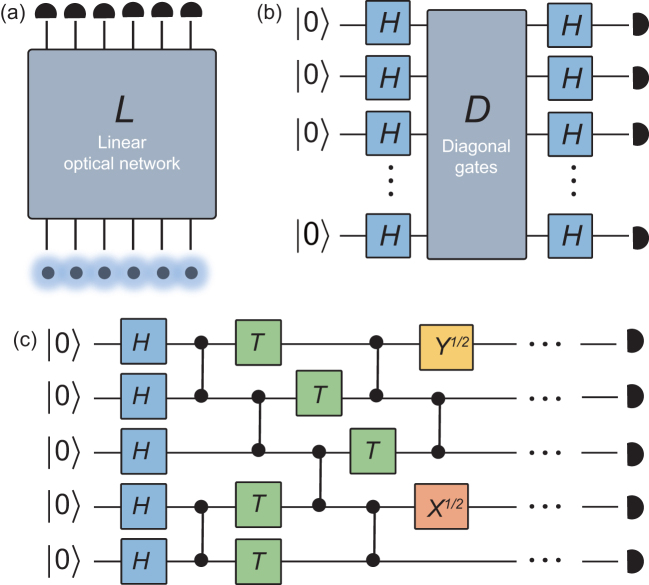
Three different approaches for achieving quantum supremacy: (a) boson sampling; (b) IQP circuits; (c) chaotic quantum circuits.

Currently, there are three popular approaches for achieving quantum supremacy (see Fig. 1), namely (i) boson sampling [4], (ii) sampling IQP (instantaneous quantum polynomial) circuits [5], and (iii) sampling chaotic quantum circuits [6]. In all of these approaches, distributions of different bit strings or photon numbers are sampled from the quantum devices. Furthermore, they all involve the assumption that classical computers are unable to efficiently determine the transition amplitudes, and/or reproduce (or approximate) the distributions of quantum devices performing these tasks, in the sense of both strong and weak simulations.

In boson sampling, single photons are injected into different modes of a linear optical network. The task is to determine the photon distributions at the output ports. The key feature of boson sampling is that the transition amplitudes are related to the permanents of complex matrices, which are in general very difficult to calculate exactly, or approximate to within an multiplicative error; these problems belong to the #P-hard complexity class. Moreover, efficient weak simulation of boson sampling is believed to be impossible, unless the polynomial hierarchy collapses to its third level. However, a recent numerical study suggests that, in order to achieve quantum supremacy with boson sampling, one needs to simultaneously generate at least 50 or more single photons, which is still highly technologically challenging.

An interesting question is whether linear optics, in the setting of boson sampling, can be applied to solve decision problems. In this case, the transition amplitudes may need to be determined to an additive error. However, we found that a large class of decision problems associated with boson sampling can be simulated by a classical computer [7], settling an open problem [8] by Aaronson and Hance.

IQP circuits represent a simplified quantum-circuit model. The initial state starts from the all-zero state, |000…0〉, followed by applying Hadamard gates to each qubit. Then, diagonal gates are applied to the qubits, followed by applying Hadamard gates to each qubit again. The argument of showing the complexity in simulating IQP circuits is similar to that of boson sampling. In fact, the complexity of boson sampling was inspired by the complexity results of IQP circuits. However, when subject to noise, IQP circuits may become classically simulable [9].

Lastly, chaotic quantum circuits refer to the class of quantum circuits where two-qubit gates are applied regularly, while single-qubit gates are applied randomly from a gate set. The output distributions of these circuits approach the so-called Porter–Thomas distribution, which is a characteristic feature of quantum chaos. Recently, there have been several numerical investigations aiming to explore the ultimate limit of classical computing in simulating low-depth quantum circuits. For the ideal cases, one needs to consider both the qubit number and circuit depth for benchmarking quantum supremacy. However, in the presence of noise, these chaotic circuits may also become classically simulable for high-depth circuits [10].

Finally, other than these three approaches, one should expect that quantum supremacy can be achieved for many practical applications, e.g. simulation of quantum chemistry or quantum machine learning. When will it happen? Only time will tell!
